# 3-Methyl-4-nitro­phenol–4-dimethyl­amino­pyridine (1/1)

**DOI:** 10.1107/S1600536812041670

**Published:** 2012-10-13

**Authors:** Srinivasan Muralidharan, Perumal Nagapandiselvi, Thothadri Srinivasan, Rengasamy Gopalakrishnan, Devadasan Velmurugan

**Affiliations:** a Department of Physics, Anna University, Chennai 600 025, India; b Centre of Advanced Study in Crystallography and Biophysics, University of Madras, Guindy Campus, Chennai 600 025, India

## Abstract

In the title adduct, C_7_H_7_NO_3_^.^C_7_H_10_N_2_, the dihedral angle betwen the benzene ring and pyridine rings is 9.60 (8)° while the nitro group attached to the benzene ring makes a dihedral angle of 21.76 (13)°. The hydroxyl O atom deviates by 0.0247 (15) Å from the plane of the benzene ring. The crystal packing features O—H⋯N hydrogen bonds.

## Related literature

For a related structure, see: Dong & Cheng (2012 ▶). 
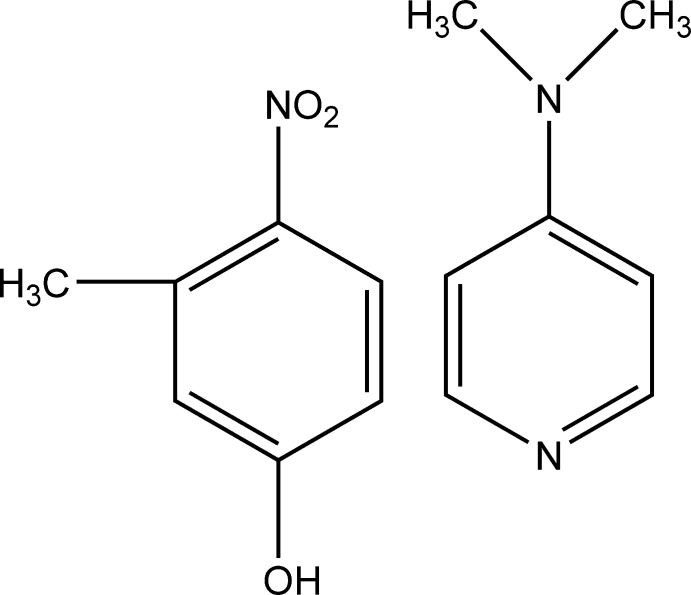


## Experimental

### 

#### Crystal data

C_7_H_7_NO_3_·C_7_H_10_N_2_
*M* *_r_* = 275.31
Monoclinic, 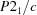

*a* = 11.4923 (9) Å
*b* = 9.8362 (8) Å
*c* = 12.7781 (10) Å
β = 103.870 (5)°
*V* = 1402.3 (2) Å^3^
*Z* = 4
Mo *K*α radiation
μ = 0.09 mm^−1^
*T* = 293 K
0.35 × 0.30 × 0.30 mm

#### Data collection

Bruker SMART APEXII area-detector diffractometer
Absorption correction: multi-scan (*SADABS*; Bruker, 2008 ▶) *T*_min_ = 0.968, *T*_max_ = 0.973
13307 measured reflections
3498 independent reflections
2469 reflections with *I* > 2σ(*I*)
*R*_int_ = 0.028

#### Refinement

*R*[*F* ^2^ > 2σ(*F* ^2^)] = 0.056
*wR*(*F* ^2^) = 0.185
*S* = 1.03
3498 reflections
184 parameters
H-atom parameters constrained
Δρ_max_ = 0.35 e Å^−3^
Δρ_min_ = −0.27 e Å^−3^

### 

Data collection: *APEX2* (Bruker, 2008 ▶); cell refinement: *SAINT* (Bruker, 2008 ▶); data reduction: *SAINT*; program(s) used to solve structure: *SHELXS97* (Sheldrick, 2008 ▶); program(s) used to refine structure: *SHELXL97* (Sheldrick, 2008 ▶); molecular graphics: *ORTEP-3* (Farrugia, 1997 ▶); software used to prepare material for publication: *SHELXL97* and *PLATON* (Spek, 2009 ▶).

## Supplementary Material

Crystal structure: contains datablock(s) global, I. DOI: 10.1107/S1600536812041670/pv2590sup1.cif

Structure factors: contains datablock(s) I. DOI: 10.1107/S1600536812041670/pv2590Isup2.hkl

Additional supplementary materials:  crystallographic information; 3D view; checkCIF report

## Figures and Tables

**Table 1:** Hydrogen-bond geometry (Å, °)

*D*—H⋯*A*	*D*—H	H⋯*A*	*D*⋯*A*	*D*—H⋯*A*
O3—H3⋯N3^i^	0.82	1.79	2.594 (2)	168

Symmetry code: (i) 
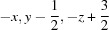
.

**Fig. 1.**
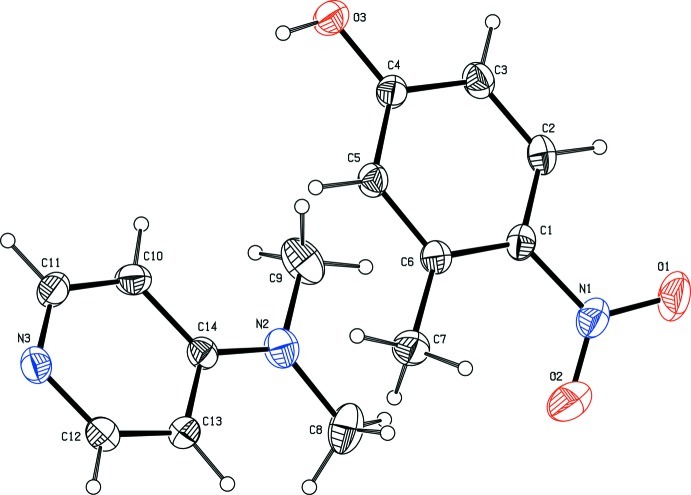
The molecular structure of the title compound, showing displacement ellipsoids drawn at the 30% probability level. H atoms are presented as small spheres of arbitrary radius.

**Fig. 2.**
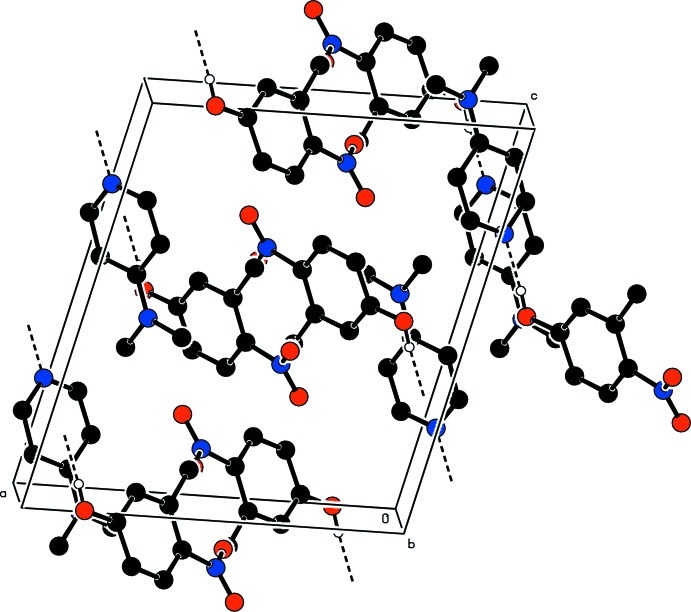
The crystal packing of the title compound viewed down c axis. H-atoms not involved in H-bonds have been excluded for clarity.

## supplementary crystallographic information

### Comment

In the molecule (fig.1),the pyridine ring (N3/C10/C11/C12/C13/C14) makes a dihedral angle of 9.60 (8)° with the phenyl ring (C1/C2/C3/C4/C5/C6) system. The oxygen atom O3 deviates by -0.0247 (15)Å from the plane of the phenyl ring. The carbon atom C7 deviates by deviates by 0.0677 (21)Å from the plane of the phenyl ring.

The nitrogen atom N1 deviates by -0.0285 (18)Å from the plane of the phenyl ring. The nitrogen atom N2 devaites by -0.0292 (18)Å from the plane of the pyridine ring. The crystal packing is stabilized by intermolecular O—H···N hydrogen bonds

### Experimental

4-Dimethylaminopyridine and 3-methyl-4-nitrophenol were taken in equimolar (1:1) ratio using acetone as solvent. The solution was filtered in a clean beaker and optimally closed. The prepared solution was kept at room temperature for two days after which crystals suitable for X-ray diffraction were obtained.

### Refinement

The hydrogen atoms were placed in calculated positions with C—H = 0.93 Å to 1.08 Å refined in the riding model with fixed isotropic displacement parameters:*U*_iso_(H) = 1.5*U*_eq_(C) for methyl group and *U*_iso_(H) = 1.2*U*_eq_(C) for other groups.

### Crystal data

**Table:**

C_7_H_7_NO_3_·C_7_H_10_N_2_	*F*(000) = 584
*M**_r_* = 275.31	*D*_x_ = 1.304 Mg m^−^^3^
Monoclinic, *P*2_1_/*c*	Mo *K*α radiation, λ = 0.71073 Å
Hall symbol: -P 2ybc	Cell parameters from 3498 reflections
*a* = 11.4923 (9) Å	θ = 1.8–28.4°
*b* = 9.8362 (8) Å	µ = 0.09 mm^−^^1^
*c* = 12.7781 (10) Å	*T* = 293 K
β = 103.870 (5)°	Block, colourless
*V* = 1402.3 (2) Å^3^	0.35 × 0.30 × 0.30 mm
*Z* = 4	

### Data collection

**Table:**

Bruker SMART APEXII area-detector diffractometer	3498 independent reflections
Radiation source: fine-focus sealed tube	2469 reflections with *I* > 2σ(*I*)
Graphite monochromator	*R*_int_ = 0.028
ω and φ scans	θ_max_ = 28.4°, θ_min_ = 1.8°
Absorption correction: multi-scan (*SADABS*; Bruker, 2008)	*h* = −15→12
*T*_min_ = 0.968, *T*_max_ = 0.973	*k* = −12→13
13307 measured reflections	*l* = −16→17

### Refinement

**Table:**

Refinement on *F*^2^	Primary atom site location: structure-invariant direct methods
Least-squares matrix: full	Secondary atom site location: difference Fourier map
*R*[*F*^2^ > 2σ(*F*^2^)] = 0.056	Hydrogen site location: inferred from neighbouring sites
*wR*(*F*^2^) = 0.185	H-atom parameters constrained
*S* = 1.03	*w* = 1/[σ^2^(*F*_o_^2^) + (0.0939*P*)^2^ + 0.361*P*] where *P* = (*F*_o_^2^ + 2*F*_c_^2^)/3
3498 reflections	(Δ/σ)_max_ = 0.001
184 parameters	Δρ_max_ = 0.35 e Å^−^^3^
0 restraints	Δρ_min_ = −0.27 e Å^−^^3^

### Special details

**Table:**

Geometry. All esds (except the esd in the dihedral angle between two l.s. planes) are estimated using the full covariance matrix. The cell esds are taken into account individually in the estimation of esds in distances, angles and torsion angles; correlations between esds in cell parameters are only used when they are defined by crystal symmetry. An approximate (isotropic) treatment of cell esds is used for estimating esds involving l.s. planes.
Refinement. Refinement of F^2^ against ALL reflections. The weighted R-factor wR and goodness of fit S are based on F^2^, conventional R-factors R are based on F, with F set to zero for negative F^2^. The threshold expression of F^2^ > 2sigma(F^2^) is used only for calculating R-factors(gt) etc. and is not relevant to the choice of reflections for refinement. R-factors based on F^2^ are statistically about twice as large as those based on F, and R- factors based on ALL data will be even larger.

### Fractional atomic coordinates and isotropic or equivalent isotropic displacement parameters (Å^2^)

**Table:**

	*x*	*y*	*z*	*U*_iso_*/*U*_eq_	
O3	0.15952 (12)	−0.10464 (16)	0.99019 (11)	0.0722 (4)	
H3	0.1231	−0.0878	0.9281	0.108*	
N3	−0.03318 (13)	0.41197 (16)	0.70694 (12)	0.0580 (4)	
N1	0.55651 (14)	0.22673 (19)	1.12638 (14)	0.0661 (4)	
O1	0.63106 (15)	0.1883 (2)	1.20437 (15)	0.0964 (6)	
N2	0.17044 (15)	0.41211 (18)	1.02568 (13)	0.0637 (4)	
C14	0.10524 (14)	0.41079 (16)	0.92229 (13)	0.0474 (4)	
C6	0.38178 (14)	0.15782 (17)	0.97841 (13)	0.0475 (4)	
C1	0.45173 (14)	0.14230 (17)	1.08398 (13)	0.0498 (4)	
C13	0.13746 (15)	0.48787 (18)	0.84064 (15)	0.0524 (4)	
H13	0.2061	0.5415	0.8567	0.063*	
C3	0.32858 (16)	−0.0365 (2)	1.12182 (14)	0.0568 (4)	
H3A	0.3113	−0.1011	1.1691	0.068*	
C10	0.00038 (16)	0.33308 (18)	0.88898 (16)	0.0569 (4)	
H10	−0.0253	0.2778	0.9381	0.068*	
C5	0.28341 (14)	0.07288 (18)	0.94833 (13)	0.0499 (4)	
H5	0.2347	0.0806	0.8791	0.060*	
C4	0.25448 (14)	−0.02386 (17)	1.01789 (13)	0.0507 (4)	
C11	−0.06391 (16)	0.33887 (19)	0.78414 (17)	0.0615 (5)	
H11	−0.1342	0.2882	0.7652	0.074*	
C2	0.42568 (15)	0.0457 (2)	1.15360 (13)	0.0558 (4)	
H2	0.4749	0.0369	1.2226	0.067*	
O2	0.56459 (19)	0.3367 (2)	1.08688 (19)	0.1206 (8)	
C12	0.06747 (16)	0.48361 (18)	0.73744 (15)	0.0560 (4)	
H12	0.0920	0.5343	0.6852	0.067*	
C7	0.40796 (19)	0.2548 (2)	0.89618 (16)	0.0650 (5)	
H7A	0.3587	0.2327	0.8264	0.098*	
H7B	0.4909	0.2478	0.8948	0.098*	
H7C	0.3910	0.3460	0.9148	0.098*	
C9	0.1410 (3)	0.3213 (3)	1.10553 (18)	0.0842 (7)	
H9A	0.0657	0.3478	1.1194	0.126*	
H9B	0.2026	0.3264	1.1711	0.126*	
H9C	0.1353	0.2297	1.0787	0.126*	
C8	0.26892 (19)	0.5044 (3)	1.06203 (19)	0.0842 (7)	
H8A	0.3380	0.4714	1.0400	0.126*	
H8B	0.2870	0.5109	1.1392	0.126*	
H8C	0.2475	0.5926	1.0311	0.126*	

### Atomic displacement parameters (Å^2^)

**Table:**

	*U*^11^	*U*^22^	*U*^33^	*U*^12^	*U*^13^	*U*^23^
O3	0.0643 (8)	0.0827 (10)	0.0624 (8)	−0.0234 (7)	0.0014 (6)	0.0170 (7)
N3	0.0535 (8)	0.0548 (8)	0.0598 (9)	0.0094 (7)	0.0020 (6)	−0.0021 (7)
N1	0.0526 (9)	0.0713 (11)	0.0682 (10)	−0.0036 (8)	0.0023 (7)	−0.0149 (8)
O1	0.0690 (10)	0.1098 (13)	0.0881 (11)	−0.0055 (9)	−0.0249 (8)	−0.0143 (10)
N2	0.0660 (10)	0.0714 (10)	0.0511 (8)	0.0051 (8)	0.0087 (7)	−0.0005 (7)
C14	0.0456 (8)	0.0435 (8)	0.0533 (9)	0.0073 (6)	0.0119 (6)	−0.0005 (7)
C6	0.0458 (8)	0.0496 (9)	0.0482 (8)	0.0043 (6)	0.0132 (6)	−0.0018 (6)
C1	0.0406 (8)	0.0544 (9)	0.0515 (9)	0.0044 (7)	0.0056 (6)	−0.0099 (7)
C13	0.0444 (8)	0.0520 (9)	0.0602 (10)	−0.0030 (7)	0.0112 (7)	0.0025 (7)
C3	0.0553 (9)	0.0649 (11)	0.0485 (9)	0.0058 (8)	0.0093 (7)	0.0111 (8)
C10	0.0567 (10)	0.0480 (9)	0.0698 (11)	−0.0029 (7)	0.0224 (8)	0.0054 (8)
C5	0.0484 (8)	0.0584 (9)	0.0399 (8)	−0.0007 (7)	0.0047 (6)	0.0034 (7)
C4	0.0459 (8)	0.0555 (9)	0.0488 (9)	−0.0016 (7)	0.0078 (6)	0.0023 (7)
C11	0.0453 (9)	0.0537 (10)	0.0813 (13)	−0.0029 (7)	0.0069 (8)	−0.0095 (9)
C2	0.0508 (9)	0.0687 (11)	0.0422 (8)	0.0103 (8)	0.0001 (7)	−0.0002 (7)
O2	0.1045 (15)	0.1048 (15)	0.1303 (17)	−0.0483 (12)	−0.0155 (12)	0.0154 (13)
C12	0.0585 (10)	0.0538 (9)	0.0563 (10)	0.0064 (8)	0.0150 (8)	0.0068 (7)
C7	0.0666 (11)	0.0673 (12)	0.0613 (11)	−0.0061 (9)	0.0157 (9)	0.0084 (9)
C9	0.1090 (18)	0.0927 (16)	0.0549 (11)	0.0277 (14)	0.0273 (11)	0.0153 (11)
C8	0.0637 (12)	0.115 (2)	0.0667 (13)	0.0000 (12)	0.0009 (10)	−0.0245 (13)

### Geometric parameters (Å, º)

**Table:**

O3—C4	1.328 (2)	C3—C4	1.401 (2)
O3—H3	0.8200	C3—H3A	0.9300
N3—C12	1.331 (2)	C10—C11	1.367 (3)
N3—C11	1.335 (3)	C10—H10	0.9300
N1—O2	1.207 (3)	C5—C4	1.396 (2)
N1—O1	1.209 (2)	C5—H5	0.9300
N1—C1	1.456 (2)	C11—H11	0.9300
N2—C14	1.354 (2)	C2—H2	0.9300
N2—C8	1.439 (3)	C12—H12	0.9300
N2—C9	1.456 (3)	C7—H7A	0.9600
C14—C10	1.404 (2)	C7—H7B	0.9600
C14—C13	1.409 (2)	C7—H7C	0.9600
C6—C5	1.384 (2)	C9—H9A	0.9600
C6—C1	1.403 (2)	C9—H9B	0.9600
C6—C7	1.502 (3)	C9—H9C	0.9600
C1—C2	1.383 (3)	C8—H8A	0.9600
C13—C12	1.371 (3)	C8—H8B	0.9600
C13—H13	0.9300	C8—H8C	0.9600
C3—C2	1.359 (2)		
			
C4—O3—H3	109.5	O3—C4—C3	118.28 (15)
C12—N3—C11	115.74 (15)	C5—C4—C3	118.74 (15)
O2—N1—O1	121.00 (19)	N3—C11—C10	124.75 (16)
O2—N1—C1	119.70 (17)	N3—C11—H11	117.6
O1—N1—C1	119.19 (19)	C10—C11—H11	117.6
C14—N2—C8	121.99 (18)	C3—C2—C1	120.37 (15)
C14—N2—C9	120.72 (18)	C3—C2—H2	119.8
C8—N2—C9	117.27 (18)	C1—C2—H2	119.8
N2—C14—C10	122.35 (17)	N3—C12—C13	124.50 (17)
N2—C14—C13	122.36 (16)	N3—C12—H12	117.8
C10—C14—C13	115.29 (15)	C13—C12—H12	117.8
C5—C6—C1	116.28 (15)	C6—C7—H7A	109.5
C5—C6—C7	118.45 (15)	C6—C7—H7B	109.5
C1—C6—C7	125.23 (16)	H7A—C7—H7B	109.5
C2—C1—C6	122.06 (15)	C6—C7—H7C	109.5
C2—C1—N1	116.10 (15)	H7A—C7—H7C	109.5
C6—C1—N1	121.85 (17)	H7B—C7—H7C	109.5
C12—C13—C14	119.88 (16)	N2—C9—H9A	109.5
C12—C13—H13	120.1	N2—C9—H9B	109.5
C14—C13—H13	120.1	H9A—C9—H9B	109.5
C2—C3—C4	119.98 (16)	N2—C9—H9C	109.5
C2—C3—H3A	120.0	H9A—C9—H9C	109.5
C4—C3—H3A	120.0	H9B—C9—H9C	109.5
C11—C10—C14	119.82 (17)	N2—C8—H8A	109.5
C11—C10—H10	120.1	N2—C8—H8B	109.5
C14—C10—H10	120.1	H8A—C8—H8B	109.5
C6—C5—C4	122.56 (14)	N2—C8—H8C	109.5
C6—C5—H5	118.7	H8A—C8—H8C	109.5
C4—C5—H5	118.7	H8B—C8—H8C	109.5
O3—C4—C5	122.98 (15)		
			
C8—N2—C14—C10	−172.07 (18)	C13—C14—C10—C11	−1.7 (2)
C9—N2—C14—C10	6.4 (3)	C1—C6—C5—C4	−0.3 (2)
C8—N2—C14—C13	7.6 (3)	C7—C6—C5—C4	177.63 (17)
C9—N2—C14—C13	−174.00 (17)	C6—C5—C4—O3	179.17 (16)
C5—C6—C1—C2	1.3 (2)	C6—C5—C4—C3	−0.7 (3)
C7—C6—C1—C2	−176.47 (17)	C2—C3—C4—O3	−179.11 (17)
C5—C6—C1—N1	−178.98 (15)	C2—C3—C4—C5	0.7 (3)
C7—C6—C1—N1	3.2 (3)	C12—N3—C11—C10	−0.4 (3)
O2—N1—C1—C2	−156.9 (2)	C14—C10—C11—N3	1.8 (3)
O1—N1—C1—C2	19.3 (3)	C4—C3—C2—C1	0.2 (3)
O2—N1—C1—C6	23.4 (3)	C6—C1—C2—C3	−1.3 (3)
O1—N1—C1—C6	−160.43 (18)	N1—C1—C2—C3	178.99 (16)
N2—C14—C13—C12	−179.41 (16)	C11—N3—C12—C13	−1.2 (3)
C10—C14—C13—C12	0.3 (2)	C14—C13—C12—N3	1.2 (3)
N2—C14—C10—C11	178.01 (17)		

### Hydrogen-bond geometry (Å, º)

**Table:**

*D*—H···*A*	*D*—H	H···*A*	*D*···*A*	*D*—H···*A*
O3—H3···N3^i^	0.82	1.79	2.594 (2)	168

Symmetry code: (i) −*x*, *y*−1/2, −*z*+3/2.
